# Serotype-Specific IgG Antibody Waning after Pneumococcal Conjugate Primary Series Vaccinations with either the 10-Valent or the 13-Valent Vaccine

**DOI:** 10.3390/vaccines6040082

**Published:** 2018-12-11

**Authors:** Els van Westen, Mirjam J. Knol, Alienke J. Wijmenga-Monsuur, Irina Tcherniaeva, Leo M. Schouls, Elisabeth A. M. Sanders, Cecile A. C. M. van Els, Guy A. M. Berbers, Nynke Y. Rots

**Affiliations:** Center for Infectious Disease Control, National Institute for Public Health and the Environment, 3720 BA Bilthoven, The Netherlands; els.van.westen@rivm.nl (E.v.W.); mirjam.knol@rivm.nl (M.J.K.); alienke.wijmenga@rivm.nl (A.J.W.-M.); Irina.Tcherniaeva@rivm.nl (I.T.); leo.schouls@rivm.nl (L.M.S.); lieke.sanders@rivm.nl (E.A.M.S.); cecile.van.els@rivm.nl (C.A.C.M.v.E.); guy.berbers@rivm.nl (G.A.M.B.)

**Keywords:** pneumococcal conjugate vaccine, PCV10, PCV13, IgG, antibody kinetics, serotype-specific

## Abstract

The two currently available ten- and thirteen-valent pneumococcal conjugate vaccines (PCV10 and PCV13) both induce serotype-specific IgG anti-polysaccharide antibodies and are effective in preventing vaccine serotype induced invasive pneumococcal disease (IPD) as well as in reducing overall vaccine-serotype carriage and transmission and thereby inducing herd protection in the whole population. IgG levels decline after vaccination and could become too low to prevent carriage acquisition and/or pneumococcal disease. We compared the levels of 10-valent (PCV10) and 13-valent (PCV13) pneumococcal vaccine induced serum IgG antibodies at multiple time points after primary vaccinations. Data from two separate studies both performed in the Netherlands in infants vaccinated at 2, 3, and 4 months of age with either PCV10 or PCV13 were compared. Antibody levels were measured at 5, 8, and 11 months of age, during the interval between the primary immunization series and the 11-months booster dose. Serotype-specific IgG levels were determined by multiplex immunoassay. Although antibody kinetics showed significant variation between serotypes and between vaccines for the majority of the 10 shared serotypes, i.e., 1, 5, 7F, 9V, 14, 18C, and 23F, antibody concentrations were sufficiently high for both vaccines, immediately after the primary series and throughout the whole period until the booster dose. In contrast, for serotypes 4 and 19F in the PCV10 group and for serotypes 4 and 6B in the PCV13 group, IgG antibody concentrations already come within reach of the frequently used seroprotection level of 0.35 μg/mL immediately after the primary series at the five month time point and/or at eight months. This paper addresses the importance of revealing differences in serotype-specific and pneumococcal vaccine-dependent IgG antibody patterns during the interval between the primary series and the booster dose, an age period with a high IPD incidence. Trial registration: www.trialregister.nl NTR3069 and NTR2316.

## 1. Introduction

*S. pneumoniae* remains a major cause of morbidity and mortality in children worldwide, specifically in children under the age of five years. The first pneumococcal conjugate vaccine (PCV) that was widely implemented for the prevention of pneumococcal disease in children contained purified bacterial capsule sugars from seven of the more than 90 identified pneumococcal serotypes conjugated to the CRM_197_ carrier protein (PCV7). PCV7 vaccination was introduced in the National Immunization Program (NIP) in the Netherlands in 2006 for all infants with vaccine doses administered at the age of 2, 3, and 4 months followed by a booster dose at the age of 11 months. As a result, IPD and carriage of pneumococcal vaccine serotypes have strongly decreased [1,2,3]. However, the beneficial effects of vaccination are eroding due to serotype replacement. In response to the increase in *S. pneumoniae* infections by non-vaccine serotypes, 10- and 13-valent vaccines (PCV10 and PCV13, respectively), licensed in 2009/2010, have replaced PCV7 in National Immunization Programs (NIP) worldwide. The two vaccines differ in the number of capsular polysaccharides included, the concentration of the polysaccharides, and the carrier protein used. In 2011, PCV7 was replaced by PCV10 in the Netherlands, while in most other countries, PCV13 was introduced.

PCVs induce serotype-specific serum IgG antibodies that are involved in protection against vaccine serotype carriage acquisition [4] and invasive pneumococcal disease (IPD) [5]. For licensure of the PCVs, an aggregated seroprotection level of 0.35 μg/mL has been used to estimate the proportion of responders likely to be protected against IPD [6]. Since then, Andrews et al. have shown that serotype-specific correlates of protection against IPD vary widely [7]. IgG antibody levels needed for the prevention of carriage also vary considerably between the serotypes [7,8]. Several studies have shown that for the prevention of pneumococcal nasopharyngeal carriage, higher antibody levels are required than for protection against IPD [8,9,10,11], but a generally accepted correlate of protection has not been identified yet. Carriage reduction is needed to decrease the transmission of pneumococcal vaccine serotypes in the population, providing herd immunity [4,8,12,13,14]. Serotype-specific antibody mediated bacterial agglutination on the mucosal surface and antibody induced complement-mediated opsonophagocytosis are two mechanisms thought to be involved in protection from carriage acquisition [15]. Mucosal IgA antibodies have mainly been implicated, but IgG antibodies, originating from the blood, can also play a role [10].

Waning serum IgG antibody levels after primary PCV vaccinations has been shown by comparing 1 month post primary series and pre-booster dose data [13,14,16]. However, there is no information on IgG antibody levels at intermediate time points. Multiple time point analysis per serotype provides a more accurate understanding of the change in antibody concentrations over time relevant for the level of protection during this interval. A fast decline in IgG antibody levels in this period could result in IgG concentrations that are insufficient to prevent IPD or pneumococcal vaccine serotype acquisition in these young and vulnerable infants.

The aim of this study was to directly compare the kinetics of the serotype-specific IgG antibody responses induced by the two currently available PCVs, PCV10 and PCV13, during the interval between the last primary series dose and the booster dose. The data will provide information relevant for the evaluation of potential serotype-specific differences between the vaccines in the prevention of IPD or carriage acquisition.

Data on IgG levels in serum samples collected at five months (1 month after the primary series), eight months, and 11 months of age (just before the booster dose) from infants immunized with PCV10 (2011/2012 trial, NTR3069) and PCV13 (2010/2011 trial, NTR2316), were compared and evaluated for each serotype.

## 2. Materials and Methods

### 2.1. Study Design

**Figure 1:**
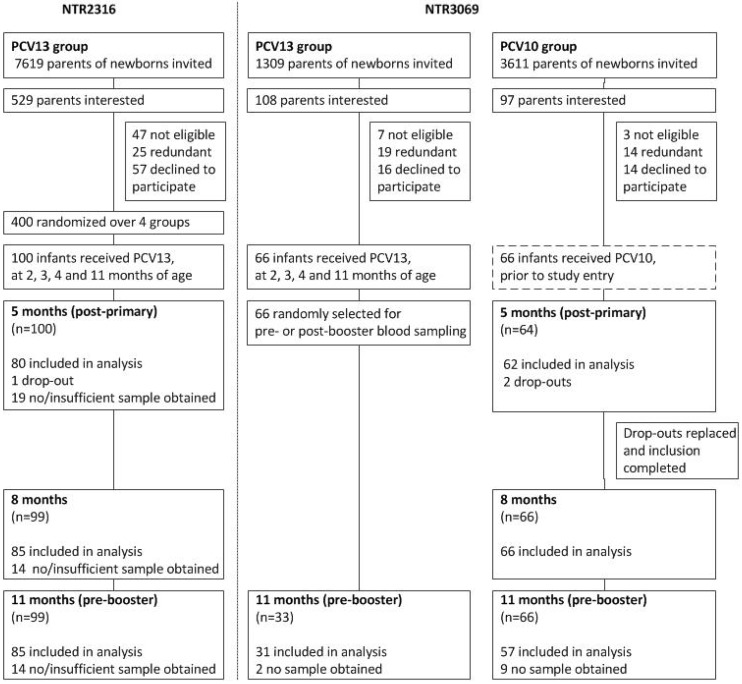
Study design and disposition of subjects in clinical trials NTR2316 and NTR3069.

Two clinical trials were performed in the Netherlands where pneumococcal serotype specific IgG antibody levels were measured at multiple intervals after the primary series vaccinations with PCV10 or PCV13. Clinical trial NTR3069 [17,18], enrolling infants from September until December 2011, compared antibody titers and plasma- and memory B cell responses around the 11-months booster dose in infants who had received PCV10 or PCV13. From the children in the PCV10 group, blood samples were collected at 5, 8, 11, and 12 months of age and for the PCV13 group at 11 and 12 month of age. In study NTR2316 [19], which enrolled infants from May until November 2010, all infants were vaccinated with PCV13, but according to four different schedules. From this trial, PCV13 induced serotype-specific antibody data measured at the age of 5, 8, and 11 months, were used (Figure 1). Different concomitant vaccines were given in both studies requiring bridging of the data, which was done by comparing PCV13-induced serotype-specific IgG levels measured at 11 months of age.

All infants in both studies were born in the Netherlands, for NTR3069 between September and December 2011, and for NTR2316 between May and December 2010, and received vaccine doses at 2, 3, and 4 months of age with a booster dose at the age of 11 months, according to the Dutch National Immunization Program.

Written informed consent was obtained from all parents and/or guardians of the study participants before participation in the study. Both studies were approved by an independent national medical ethics committee and performed in accordance with Good Clinical Practice, which includes provisions of the Declaration of Helsinki. For trial NTR2316, approval was given on 24 March 2010 by the Central Committee on Research involving human subjects (CCMO) and for NTR3069 from The Medical Ethical Committee Noord-Holland on the 7 September 2011.

### 2.2. Vaccines

PCV10 (Synflorix^®^, GSK, Wavre, Belgium) contains 1 μg polysaccharide (PS) for serotypes 1, 5, 6B, 7F, 9V, 14, and 23 F and 3 μg for serotypes 4, 18C, and 19F. All polysaccharides were conjugated to protein D from *Haemophilus influenzae* type b, with the exceptions of serotype 18C (conjugated to tetanus toxoid) and 19F (conjugated to diphtheria toxoid). PCV13 (Prevenar13^®^, Pfizer, Tadworth, UK) contains the same serotypes as PCV10 with the addition of serotypes 3, 6A, and 19A, and all serotypes were conjugated to CRM_197_ at a concentration of 2.2 μg PS, except for serotype 6B (4.4 μg).

Infants in trial NTR3069 concomitantly received Infanrix-hexa (DTaP-IPV-HBV-Hib) (GSK, Wavre, Belgium), directed against infections with diphtheria, tetanus, pertussis, polio, *Haemophilus influenzae* type b, and hepatitis B. Infants in trial NTR2316 concomitantly received Pediacel (DTaP-IPV-Hib, Sanofi Pasteur, Lyon, France), protecting against the same pathogens, but without hepatitis B. In study NTR3069, PCV10 was administered during routine visits to well-baby clinics, while PCV13 was given during home visits by study nurses in both studies.

### 2.3. Blood Collection and Laboratory Analyses

Blood was collected by heel sticks at 5 and 8 month of age and heel stick or venipuncture at 11 month of age. Serum was separated and stored at −20 °C until use. Serotype-specific IgG levels against the 13 vaccine-serotypes were analyzed using the multiplex immunoassay (MIA), as described by Elberse et al. [20]. Samples of both trials were analyzed around the same time using the same beads.

### 2.4. Statistical Analyses

All analyses were performed on log-transformed IgG levels. Geometric mean concentrations (GMC) and 95% confidence intervals were calculated. To confirm comparability between the two trials, serotype-specific IgG GMCs at 11 month of age from the PCV13 groups of trial NTR3069 and trial NTR2316 were compared for all 13 serotypes. GMCs were considered comparable if the 95% confidence interval of the GMC ratio did not exceed the predefined upper- and lower equivalence margins of 0.5 and 2.0, respectively. To assess whether the kinetics of the serotype-specific IgG levels differed between PCV10 and PCV13, a GEE analysis with exchangeable correlation structure was performed. We included the vaccine (PCV10 or PCV13), age at blood sampling (as a continuous variable), and the interaction between vaccine and age at blood sampling in the model. The variation in PCV10 and PCV13 induced antibody titers over time (the slope) was expressed as the GMC ratio per month. In addition, we present the direction (negative or positive) and p-value of the interaction between vaccine and age at blood sampling to indicate how and whether the trend over time differed between the vaccines. All data were analyzed using SPSS 22.0 and GraphPad Prism 6 software.

## 3. Results

### 3.1. Study Population

Presented serological data originated from two different clinical trials. Figure 1 depicts the participant disposition of the part of both trials that is relevant for this analysis (Figure 1). Baseline characteristics were similar, but infants in the PCV10 group were 0.1–0.4 months older at the 2, 3, and 4 months vaccination moments and the 5-months blood sampling time point, and were 0.5 months younger at the 11-months blood sampling time point when compared to infants from the PCV13 group of the NTR2316 trial (Table S1).

### 3.2. Comparison of IgG Levels at 11 Months of Age, between PCV13 Groups from Both Trials

**Figure 2:**
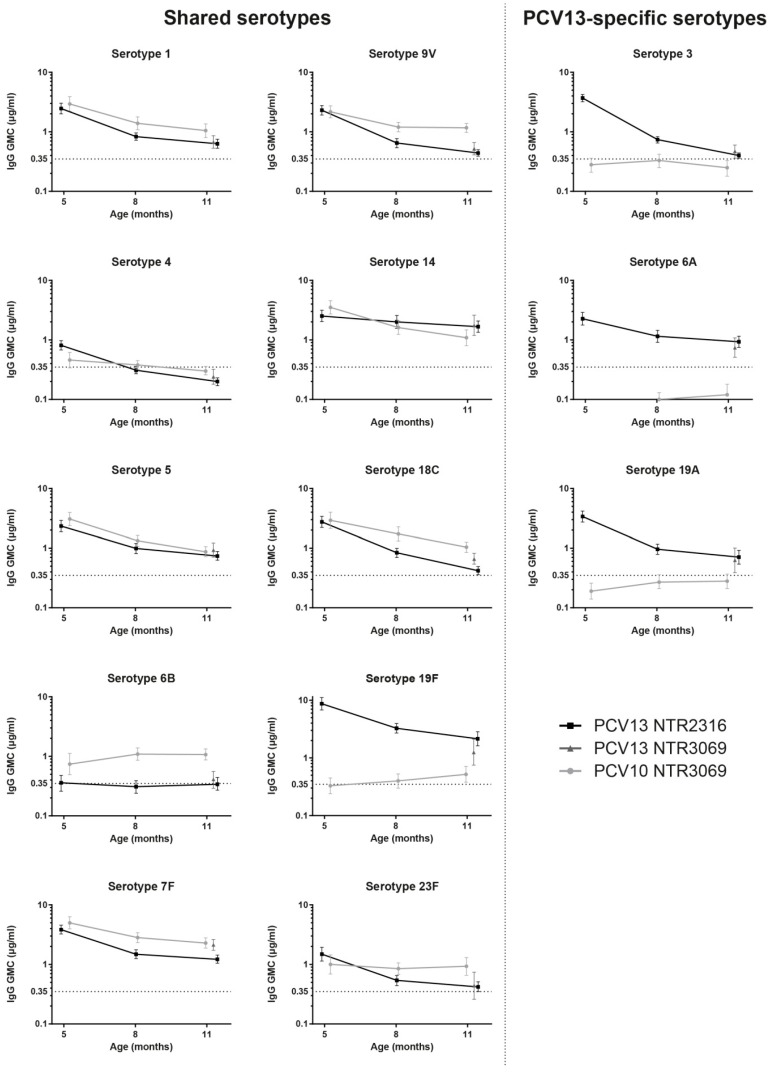
Serotype-specific IgG concentrations at 5, 8, and 11 months of age. IgG levels were expressed as geometric mean concentrations (GMCs) with 95% CIs of infants vaccinated with PCV10 or PCV13. Age at blood sampling was set as a continuous variable. For serotype 6A, the GMC was 0.06 μg/mL at 5 months in the PCV10 group.

Comparability of the antibody data between the two trials was investigated by comparing serum IgG GMCs of PCV13 vaccinated infants at 11 months of age. IgG levels for 10 out of 13 serotypes were equivalent; only for serotypes 7F, 18C, and 19F equivalence criteria were not met (Figure 2). IgG antibody levels for serotypes 7F and 18C were significantly higher and for 19F were significantly lower in PCV13-vaccinated infants included in 2011/2012 (NTR3069 study), compared to the PCV13-vaccinated counterparts included in 2010/2011 (NTR2316 study). These differences in absolute antibody levels for 7F, 18C, and 19F between the two studies will be taken into account when antibody kinetics for the two vaccines are discussed.

### 3.3. Kinetics of IgG Responses to the 10 Shared-Serotypes Induced by PCV10 and PCV13

One month after the primary series at five months of age, antibody GMCs were similar for both vaccines for the 10 shared-serotypes, except for serotypes 4 and 19F for which they were higher, and for 6B for which they were lower after PCV13 when compared with the PCV10 vaccination. For most of the serotypes, specific IgG antibody levels declined over time between five and 11 months of age for both groups (Figure 2, Table 1). Significant differences in the waning of antibodies between the PCV10 and PCV13 groups were seen for seven of the ten shared-serotypes during the six month interval (Table 1). For five out of these seven serotypes (4, 9V, 18C, 19F, and 23F), a significant negative interaction was shown, indicating an overall faster antibody decline over time in the PCV13 group when compared with the PCV10 group. Serotype 23F specific IgG antibody levels did not change over time after PCV10 vaccination while they decreased after immunization with PCV13 (Figure 2, Table 1). For serotypes 5 and 14, a positive interaction was found, indicating a faster decline for PCV10-induced antibodies. In addition, serotype 14-specific IgG GMCs were higher in the PCV13 group when compared with the PCV10 group at 11 months of age (Figure 2, Table 1). For serotypes 1, 6B, and 7F, no interaction was found. For serotypes 1 and 7F, this meant that antibody levels waned at an equal speed in both groups, whereas for serotype 6B, the antibody levels did not change over time for both groups. Notably, for serotype 4 (PCV10 and PCV13) 6B, (PCV13), and 19F (PCV10), vaccine induced IgG antibody GMCs just above or below the 0.35 μg/mL threshold were measured during most of the six month interval.

### 3.4. PCV13-Specific Serotypes

IgG levels directed against the additional serotypes of PCV13 (3, 6A, and 19A) were high at five months of age and declined over time, similar to the IgG antibodies induced by the shared-serotypes in the PCV13 immunized infants (Figure 2). As expected, infants that received the PCV10 vaccine, without serotypes 3, 6A, and 19A, had low serum IgG antibody levels directed against these serotypes (Figure 2).

## 4. Discussion

Infant vaccination schedules in general contain an interval of at least six months between the primary series of vaccine doses and the booster dose which is needed to establish an optimal long-term immune memory response. However, for pneumococcus, this interval overlaps with the peak incidence of IPD. Protection against vaccine type IPD during this period thus requires sufficiently high antibody concentrations immediately after the primary PCV doses with limited decline.

The aim of this study was to optimally compare the levels and kinetics of pneumococcal serotype-specific IgG responses during the seven month interval between the last primary series dose at four months of age and the 11-month booster dose in Dutch infants vaccinated with either PCV10 or PCV13. To allow a more detailed analysis, the infants’ blood samples were obtained not only at five and 11 months of age, but also at an additional intermediate eight month time point. Inclusion of the intermediate time point sample had added value, albeit modest, in that it revealed for serotypes with declining antibody levels that the decay was faster in the first half of the interval than in the second half.

The main finding of our study is that for seven of the 10 shared serotypes, 1, 5, 7F, 9V, 14, 18C, and 23F, both vaccines induced antibody concentrations well above the frequently used seroprotection level of 0.35 μg/mL, immediately after the primary series and throughout the whole period until the first booster dose. Overall, three doses of PCV10 or PCV13 resulted in similar IgG antibody levels for these serotypes one month after the primary series doses. The subsequent decline was either similar irrespective of the vaccine used (serotype 1, 7F), faster in the PCV10 group (serotype 5, 14), or faster in the PCV13 group (serotype 9V, 18C, and 23F). Thus, although antibody kinetics showed significant variation between serotypes and between vaccines for the majority of the shared serotypes, both vaccines seem to provide sufficient protection during the interval.

In contrast, IgG antibody concentrations for serotypes 4 and 19F in the PCV10 group and for serotypes 4 and 6B in the PCV13 group were low and already in the range of the seroprotection level immediately after the primary series or at eight months. In the Netherlands, serotype 4- and 6B-related carriage and IPD in children has been very low during the past few years, therefore the impact of low antibody concentrations for this serotype is expected to be limited. Serotype 19F is still circulating and has caused a few IPD cases [21].

To evaluate the risk of low serotype-specific seroprevalence, not only the circulation of the serotypes but also the timing of the booster dose has to be taken into account. For some additional serotypes, e.g., 9V, 18C, and 23F in the PCV13 group, antibody concentrations were low at the 11 month time point. Assuming that a further decline would occur, a booster dose at a later time point than 11 months could be associated with an elevated risk for pneumococcal disease for these serotypes.

Vaccine-induced serotype 6B-specific IgG antibody levels did not show a decline during the interval for both vaccine groups, which is in line with the data reported by Vesikari et al. and Kieninger et al. who also reported stable IgG antibody levels against serotype 6B in PCV10 or PCV13 vaccinated infants, respectively [13,14]. A possible explanation for this phenomenon, continued exposure to serotype 6B pneumococci, seems unlikely in view of the low 6B prevalence data [21,22]. PCV13 induced low serotype 6B-specific antibody concentrations, also found in other studies, showed weak immunogenicity of serotype 6B after primary PCV vaccinations [23,24]. Despite containing less 6B polysaccharide than PCV13, the PCV10 vaccination induced higher levels of serotype 6B-specific IgG antibodies. Vice versa, while PCV10 contains more 19F polysaccharide than PCV13, PCV10-induced IgG antibody levels against serotype 19F were low at all time points and were significantly lower compared to the PCV13 group. These discrepancies between antigen content and immunogenicity of products could be related to differences in carrier proteins or the manufacturing process [25].

A limitation of this study was that the PCV10 and PCV13 data were obtained from two different clinical trials with small differences in age at vaccination and blood collection, the use of different concomitant vaccines, and a difference in inclusion year and season, yet both studies were performed in a period with low PCV7 vaccine serotype carriage. The impact of the disparities in demographics between the studies appeared limited, since similar PCV13 induced IgG levels in 11 month old children were measured in the two trials for 10 out of 13 serotypes. Only IgG GMCs for serotypes 7F, 18C, and 19F were significantly different between the PCV13 groups of both studies. For these three serotypes, the actual difference in PCV10 and PCV13 immunogenicity might in fact be smaller, since the antibody levels of the PCV13 group in the NTR3069 study were more comparable to the PCV10 group in the same NTR3069 study than the PCV13 data from the NTR2316 study.

Children in the NTR3069 study (PCV10 group) were immunized with the hexavalent DTaP-IPV-Hib-HBV vaccine whereas children in the NTR2316 study (PCV13 group) received the pentavalent DTaP-IPV-Hib vaccine without hepatitis B. Differences in interference by DTaP-IPV-Hib-HBV and DTaP-IPV-Hib have been described previously in a study conducted in the Netherlands [26]. The authors only showed interference for serotype 18C with higher antibody levels generated when DTaP-IPV-Hib-HBV was used, which is consistent with the data presented here [26]. However, interference for serotype 19F was not shown [26].

The strength of this study is the comparison of the early serological response to PCV10 and PCV13, administered using the same immunization schedule, performed in the same geographical area with the same epidemiological background. Moreover, antibody analysis has been performed in the same lab using the same assay.

In conclusion, our detailed comparative analysis of PCV10 or PCV13 induced pneumococcal serotype-specific antibody levels in the period between the last primary series dose and the booster dose, an age period with a high IPD incidence, indicated sufficiently high IgG antibody concentrations throughout the entire interval for most shared serotypes. In general, antibody levels declined faster for serotypes 4, 9V, 19F, and 23F in the PCV13-vaccinated group, and for serotypes 5 and 14 in the PCV10 group. The intermediate time point, to capture more precisely the declining patterns, had only modest added value and can therefore be safely left out of future studies. Only for serotypes 4, 6B, and 19F IgG antibody levels could be insufficient in the studied interval, depending on the vaccine used. Whether this poses a risk relates to the circulation of serotypes, which is highly variable between countries [27]. For the choice of a PCV product, combined country-specific serotype prevalence and serotype-specific immunogenicity should be taken into account.

## Figures and Tables

**Table 1:** Comparison of PCV10 and PCV13 antibody levels over time, with exact age at blood sampling as continuous variable and exchangeable correlation structure.

Serotype	Trend Over Time				Interaction	
	PCV10		PCV13			
	GMC Ratio per Month ^a^	*p*-Value	GMC Ratio per Month ^a^	*p*-Value	Direction ^b^	*p*-Value
1	0.82	<0.001	0.82	<0.001	Negative	0.811
4	0.93	<0.001	0.81	<0.001	Negative	<0.001
5	0.79	<0.001	0.84	<0.001	Positive	0.019
6B	1.07	0.035	1.00	0.906	Negative	0.106
7F	0.86	<0.001	0.84	<0.001	Negative	0.252
9V	0.89	<0.001	0.78	<0.001	Negative	<0.001
14	0.81	<0.001	0.94	0.010	Positive	<0.001
18C	0.83	<0.001	0.76	<0.001	Negative	<0.001
19F	1.10	0.005	0.81	<0.001	Negative	<0.001
23F	0.99	0.824	0.83	<0.001	Negative	<0.001
3	0.98	0.519	0.72	<0.001	Negative	<0.001
6A	1.11	0.009	0.88	<0.001	Negative	<0.001
19A	1.08	0.015	0.79	<0.001	Negative	<0.001

^a^ Slope of the GMCs in time; ^b^ Positive interaction means: PCV13 response decreases less over time than PCV10; ^b^ Negative interaction means: PCV13 response decreases more over time than PCV10.

## Supplementary Materials

The following are available online at http://www.mdpi.com/2076-393X/6/4/82/s1, Table S1: baseline characteristics of study participants.
