# Supplementary material for: Serotype-Specific IgG Antibody Waning after Pneumococcal Conjugate Primary Series Vaccinations with either the 10-Valent or the 13-Valent Vaccine
Source: Vaccines (Basel). 2018 Dec 11;6(4):82. doi: 10.3390/vaccines6040082 (PMC6313931; doi:10.3390/vaccines6040082)
Supplement: Supplementary file 1 [file vaccines-06-00082-s001.pdf]

Table S1: Baseline characteristics of study participants.

	NTR2316	NTR3069	
	**PCV13**	**PCV13**	**PCV10**
	**(n=100)**	**(n=33)**	**(n=66)**
Male sex, %	55.0	60.6	50.0
Date of birth, median (min-max)	Aug 2010 (May-Nov)	Sept 2011 (Sept-Sept)	Nov 2011 (Oct-Dec)
Birth weight in grams, mean (SD)	3631 (458)	3626 (496)	3636 (471)
Age in months, mean (SD)			
At 2 months vaccination	1.9 (0.12)	1.9 (0.11)	2.0 (0.17)*
At 3 months vaccination	2.9 (0.14)	2.9 (0.12)	3.2 (0.25)*
At 4 months vaccination	3.9 (0.16)	3.9 (0.12)	4.3 (0.31)*
At 5 months blood sampling	4.9 (0.18)	-	5.3 (0.34)*
At 8 months blood sampling	8.1 (0.13)	-	8.1 (0.12)
At 11 months blood sampling	11.5 (0.16)	11.3 (0.12)*	11.0 (0.27)*

* p value < 0.05 of the PCV10 and PCV13 groups of NTR3069 both compared to their counterparts of the PCV13 group of trial NTR2316
